# Aggregation potency and proinflammatory effects of SARS-CoV-2 proteins

**DOI:** 10.1038/s41598-025-10013-1

**Published:** 2025-08-04

**Authors:** Monica Costa, Da-Wei Wang, Kai-Dong Zhao, Lin Yuan, Anita Krisko, Jia-Yi Li, Tiago Outeiro, Wen Li

**Affiliations:** 1 Department of Experimental Neurodegeneration, Center for Biostructural Imaging of Neurodegeneration, University Medical Center Göttingen, 37073 Göttingen, Germany; 2 Laboratory of Research in Parkinson’s and Related Disorders, Key Laboratory of Major Chronic Diseases of Nervous System of Liaoning Province, Health Sciences Institute, China Medical University, Shenyang, 110122 China; 3 Neural Plasticity and Repair Unit, Department of Experimental Medical Science, Lund University, 22184 Lund, Sweden; 4 Max Planck Institute for Multidisciplinary Sciences, 37075 Göttingen, Germany; 5 Translational and Clinical Research Institute, Faculty of Medical Sciences, Newcastle University, Framlington Place, Newcastle Upon Tyne, NE2 4HH UK; 6 Scientific Employee With an Honorary Contract at Deutsches Zentrum Für Neurodegenerative Erkrankungen (DZNE), 37073 Göttingen, Germany

**Keywords:** SARS-CoV-2 protein, Neurodegeneration, Coronavirus disease 2019, Inflammation, Protein aggregation, Cell biology, Microbiology, Neuroscience, Diseases

## Abstract

Coronavirus disease 2019 (COVID-19), caused by severe acute respiratory syndrome coronavirus 2 (SARS-CoV-2) infection, is primarily known as a respiratory disease. The continued study of the disease has shown that long-term COVID-19 symptoms include persisting effects of the virus on the brain when the infection is over, possibly even leading to neurodegeneration. However, the exact mechanisms of nervous system damage induced by SARS-CoV-2 are still unclear. In this study, we focused on two possibly shared pathways of SARS-CoV-2-induced neural dysfunction and neurodegeneration: protein aggregation, which is associated with impaired protein clearance, and inflammatory responses, which involve a hyper-active immune status. We observed distinct expression and distribution patterns of ten SARS-CoV-2 proteins in the two cell lines, meanwhile forming aggregation puncta and inducing pro-inflammatory responses. We found that the ER stress was induced and that the autophagy-lysosome pathway was inhibited upon viral protein expression. Boosting autophagy function attenuated protein aggregation, suggesting that modulation of autophagy might be a valid strategy for inhibiting cytotoxic effects of SARS-CoV- 2 proteins. Our study provides potential explanations of SARS-CoV-2-induced cell damage, based on shared cellular mechanisms and furthermore, suggests that modulation of proteostasis may serve as therapeutic strategies for preventing long-lasting SARS-CoV-2 cytotoxic effects.

## Introduction

Nearly four years ago, the global-wide outbreak of the coronavirus disease 2019 (COVID-19) has caused severe threats to public health around the world, with a continuing impact until today^1,2^. COVID-19 is caused by the infection by severe acute respiratory syndrome coronavirus 2 (SARS-CoV-2), which attacks primarily the respiratory system^3^. Research on COVID-19 patients has revealed neurological symptoms including headache, anosmia, seizure, etc., especially during post-COVID period, suggesting a prolonged effect after SARS-CoV-2 infection in the nervous system^4,5^. It is still unknown whether the neurological symptoms of COVID-19 result from indirect effects of systematic immune activation or the direct entry of SARS-CoV-2 viral particles and proteins into brain cells, causing sequential cellular deficits including neuroinflammation and neuronal damage.

There are speculations that the neurological symptoms in COVID-19 may be a secondary effect of systematic immune activation, as both post-mortem analyses and brain single cell sequencing showed the presence of CD8 + T cells and macrophages in perivascular regions and infiltration in the central nervous system (CNS)^6,7^. Meanwhile, activated microglia in COVID-19 brain contains viral RNA. Multiorgan and neuropathological analysis have also revealed viral RNA/protein contents in the brains of COVID-19 patients^7–9^. This suggests the tropism of SARS-CoV-2 to the neuronal network and, therefore, it is reasonable to suspect that the neurological symptoms associated with SARS-CoV-2 infection could be a result of direct harm from viral particles or proteins.

SARS-CoV-2 is a member of the coronavirus family and, like most of the typical RNA viruses, SARS-CoV-2 infects and replicates by forming protein interactions with the recipient cell machinery including subcellular organelles such as endoplasmic reticulum (ER)^10,11^. The SARS-CoV-2 single-stranded RNA genome is around 30 kb, encoding mainly three types of confirmed proteins^12^. From the 5’ end of the genome, ORF1a and ORF1b are translated as polyproteins, which are further cleaved into 16 nonstructural proteins (NSPs). The 3’ end subgenomic regions encode conservative proteins including spike (S), envelope (E), membrane (M) and nucleocapsid (N)^13,14^. The rest of the genome encodes for accessory proteins of ORF3a, ORF3b, ORF6, ORF7a, ORF7b, ORF8, ORF9b and ORF10^15^.

SARS-CoV-2 viral proteins can be neuroinvasive similar to full viruses, as they were also found in postmortem brains of COVID-19 patients^16^. This implies the possibility that SARS-CoV-2 viral proteins may have direct cytotoxic effects in the brain, similar to the immune system and kidney. However, the molecular mechanisms of neuronal dysfunction induced by SARS-CoV-2 proteins still remain unknown.

From the genetic correlation analyses, COVID-19 hospitalization is positively correlated with the ratio of neurodegenerative diseases, such as Alzheimer’s disease (AD)^17^. The pathogenic hallmarks of SARS-CoV-2 infection may overlap partially with neurodegeneration, especially in the aspects of neuroinflammation and protein dys-homeostasis. In fact, ORF6 and ORF10 show amyloidogenic properties and neurotoxicity in neuroblastoma cells^18^. Based on computational algorithm predictions, NSPs possess aggregation-prone regions, and may form toxic amyloid assemblies^19^. On the other hand, the envelope protein of SARS-CoV-2 was shown to activate both astrocytes and microglia in various brain regions, inducing depression-like behavior in rodents^20^. The spike glycoprotein of SARS-CoV-2 promotes NLRP3 inflammasome formation by activating NF-κB signaling^21^. Therefore, SARS-CoV-2 proteins may induce neurological disturbances both by facilitating amyloidogenic processes in neurons and aggravating glial activation.

In the present study, based on their in silico predicted propensity to aggregate, we studied eight non-structural proteins of SARS-CoV-2, including NSP4, NSP6, NSP7 (non-aggregating control), ORF3a, ORF6, ORF7a, ORF7b, ORF10, and the structural proteins CoV E and CoV M. By expressing these proteins in distinct cell types (HEK293T and microglia), we aimed to investigate the subcellular distribution, aggregation propensity, and pro-inflammatory effects of these proteins, in order to gain insight into the cellular and molecular mechanisms of SARS-CoV-2 protein induced cellular dysfunction, which could constitute a possible explanation for COVID-19 associated neurological sequelae, and may inform on novel targets for therapeutic intervention.

## Results

### Expression of the SARS-CoV-2 proteins in HEK293T cells

**Fig. 1:**
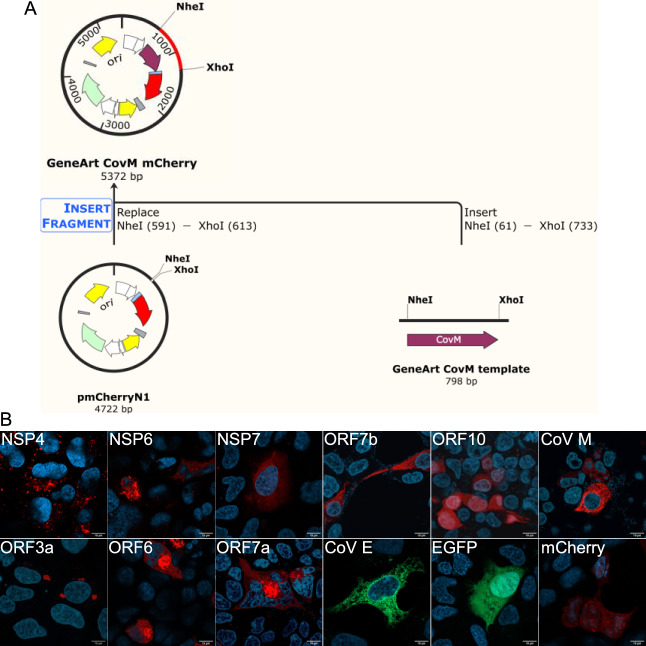
Expression of the 10 Sars-CoV-2 fluorescent fused proteins in HEK293T cell line. A: Geneart construction of vectors expressing Sars-CoV-2 proteins, using Cov M as an example. B: Transfection of HEK293T cells with Sars-CoV-2 fused proteins with mCherry fluorescence protein (NSP4, NSP6, NSP7, ORF3a, ORF6, ORF7a, ORF7b, ORF10, Cov M) and with EGFP fluorescence protein (Cov E). Representative images from three different experiments (n = 3). Scale bars: 10 μm.

The expression of the 10 SARS-CoV-2 proteins was initially analysed in human embryonic kidney cell line (HEK293T). In this study, we transfected HEK293T cells with plasmids encoding for the viral proteins NSP4, NSP6, ORF3a, ORF6, ORF7a, ORF7b, ORF10, or CoV M fused to mCherry (Fig. 1 A), or CoV E fused to EGFP protein. The viral proteins were selected based on their predicted aggregation potency, and were compared with NSP7, selected as a non-aggregating control^14^ (Supplementary Fig. 1). All constructs resulted in expression of the desired proteins fused to the respective fluorescent protein (Fig. 1 B).

### SARS-CoV-2 proteins form inclusions in HEK293T cells

**Fig. 2:**
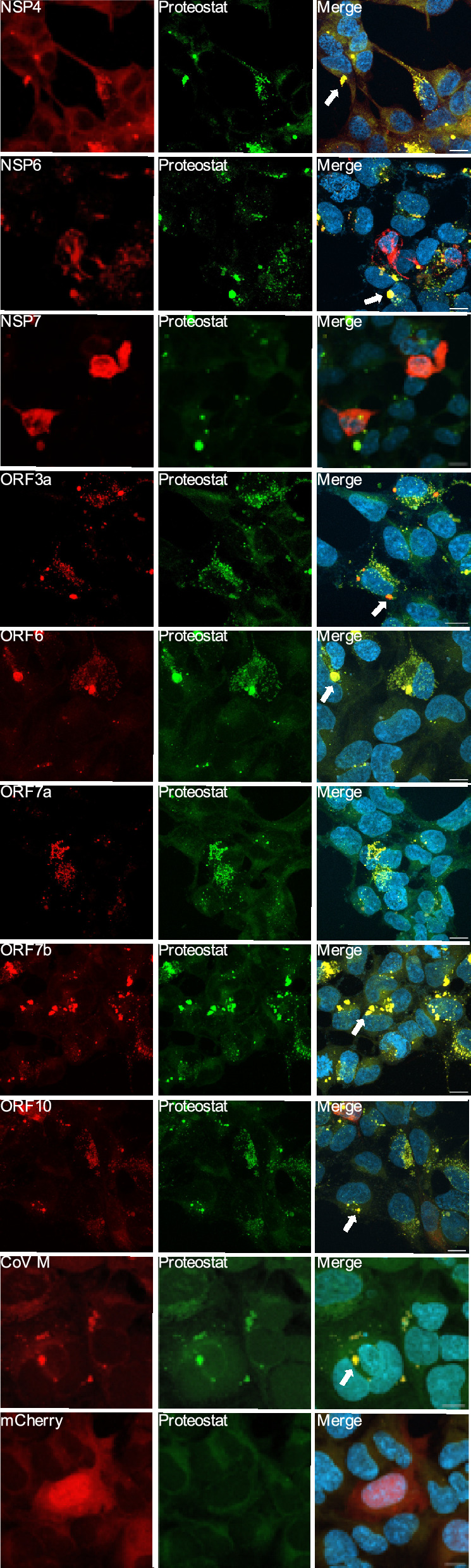
Aggregation propensity of selected Sars-Cov-2 proteins using Proteostat dye staining. HEK293T cells transfected with 9 Sars-CoV-2 constructs fused with mCherry (red) and stained with Proteostat dye (green) were shown. The formation of aggregates is indicated by colocalization signal in yellow. Puncta-patterned deposition is pointed out with white arrow. Confocal imaging, 63X objective (n = 3, maximum Z projection from approx. 30 zstacks). Scale bar:10 µm.

Several SARS-CoV-2 proteins were previously described to contain aggregation-prone regions (APR) based on analyses using four different bioinformatic tools: Pasta2.0, CamSol, Amylogram and Tango^22^, similar to proteins associated with neurodegenerative conditions (Supplementary Fig. 1). Therefore, we first investigated the aggregation potential of the selected SARS-CoV-2 proteins predicted to have a high aggregation propensity^22^, as well as NSP7, predicted not to aggregate, as a control (Supplementary Fig. 1). Twenty-four hours after transfection, we used the Proteostat Dye to assess the presence of amyloid-like structures (Fig. 2, Table 1). NSP4, NSP6, ORF3a, ORF6, ORF7b, ORF10 and CoV M formed large Proteostat-positive inclusions. ORF7a formed small dot-like aggregates that were positive for the Proteostat-dye, while NSP7 and mCherry (used as a control) formed no inclusions, as predicted (Fig. 2, Table 1).

**Table 1:** Expression and effects SARS-CoV2 proteins in HEK293T and BV2 cells.

Protein	Expression in HEK	Aggregation in HEK	Expression in BV2	Proinflammatory effects in BV2		
				**TNF-α**	**IL-6**	**IL-1β**
**NSP4**	-	+	-	N/A	N/A	N/A
**NSP6**	-	+	-	N/A	N/A	N/A
**NSP7**	-	-	-	N/A	N/A	N/A
**ORF3a**	-	+	-	N/A	N/A	N/A
**ORF6**	+	+	+	+	+	-
**ORF7a**	+	+	+	+	+	-
**ORF7b**	+	+	+	+	+	-
**ORF10**	+	+	+	+	+	-
**CoV E**	+	+	-	N/A	N/A	N/A
**CoV M**	+	-	-	N/A	N/A	N/A

Table 1 shows the summarized information of aggregation potency and proinflammatory reaction of various SARS-CoV2 proteins. “+”, aggregation or inflammation present; “-”, aggregation or inflammation absent; N/A, construct not expressed in BV2 cells.

### SARS-CoV-2 proteins induce inflammasome-independent proinflammatory effects in BV2 cells

**Fig. 3:**
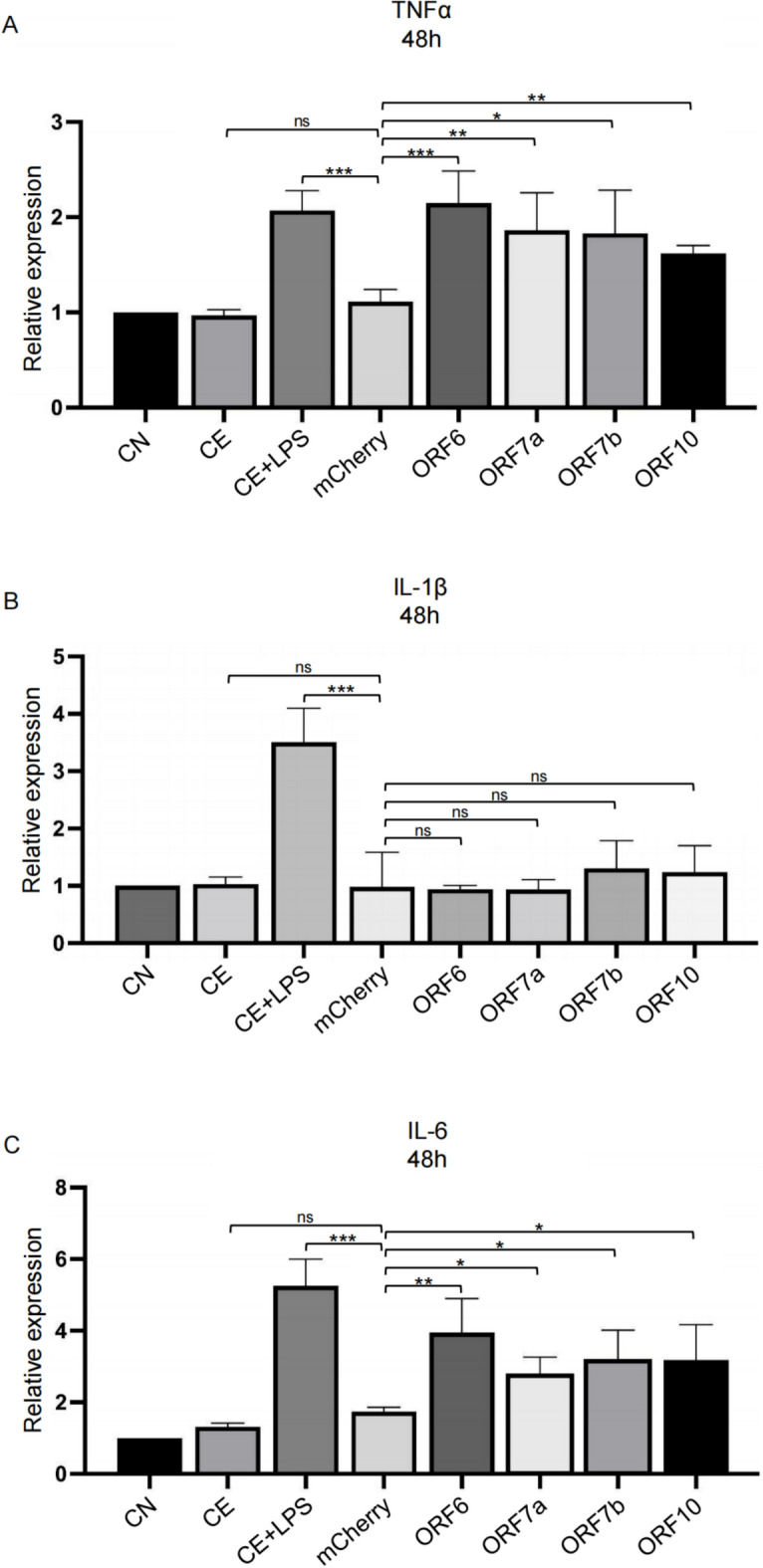
Production of proinflammatory cytokines induced by selected Sars-CoV-2 proteins. Quantification of mRNA relative expression of TNF-α (**A**), IL-1β (**B**), IL-6 (**C**) in microglia transfected with ORF6, ORF7a, ORF7b or ORF10 constructs. Data from n = 3 independent experiments. CN, control group; CE control treated with electroporation; CE + LPS, control treated with electroporation and LPS treatment; ns, not significant; *p ≤ 0.05, **p ≤ 0.01 and ***p ≤ 0.001.

SARS-CoV-2 has been known for its immune-activating properties, that have been broadly discussed and recognized as an important trigger of neurological pathology in cases of long-COVID-19. Thus, we employed the BV2 microglia cell line as a model of CNS immune activation to analyse the proinflammatory effects of the selected SARS-CoV-2 proteins. Of all the SARS-CoV-2 proteins tested in HEK293T cells, only four (ORF6, ORF7a, ORF7b and ORF10) could be efficiently transfected in BV2 microglial cells (Supplementary Fig. 2). After transfection, the levels of mRNA of selected inflammatory cytokines were measured. The mRNA levels of TNF-α and IL-6, typical cytokines indicating microglial activation, increased significantly 48 h after expression of each of the four proteins (Fig. 3 A, C). ORF6 induced the highest level of cytokine expression. On the contrary, IL-1β was not altered between viral protein groups and mCherry control, suggesting that microglia activation may take place via an NF-κB-related but inflammasome-independent pathway (Fig. 3 B)^23^.

### Subcellular distribution of SARS-CoV-2 proteins in HEK293T cells

Different SARS-CoV-2 proteins exhibited distinct aggregation patterns and proinflammatory responses in the cells tested, as shown above. In order to investigate the underlying mechanisms of the effects observed, we first examined the subcellular localization of the SARS-CoV-2 viral proteins, in order to establish specific intracellular effects on different organelles, such as ER, mitochondria and lysosomes.

**Fig. 4:**
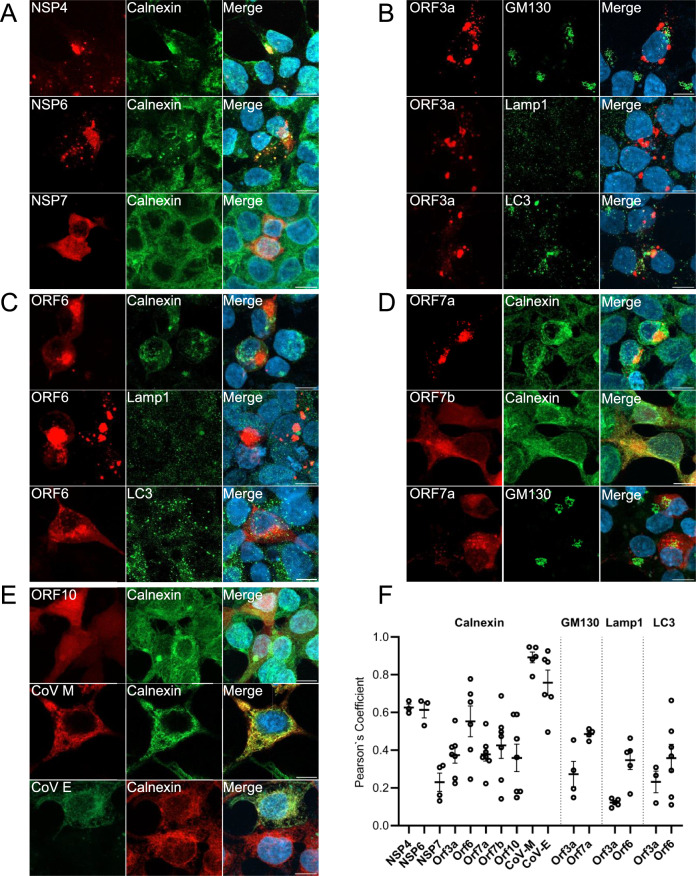
Subcellular localization of Sars-CoV-2 proteins in HEK293T cells. Co-localization of Sars-CoV-2 proteins tagged with mCherry in transfected HEK293T cells with organelles markers: Calnexin (ER marker), GM130 (Golgi marker), Lamp1 (lysosomal marker) and LC3 (autophagosome marker). A: NSP4, NSP6, NSP7 (red) and ER (green); B: ORF3a (red) and Golgi, autophagosome and lysosome (green); C: ORF6 (red) and ER, autophagosome and lysosome (green); D: ORF7a, ORF7b (red) and ER, Golgi (green); E: ER (green) and ORF10, CoV M (red), together with CoV E (green) and ER (red). F: colocalization quantification with analysis of Pearson`s coefficient for each Sars-CoV-2 protein respectively. Statistical analysis (average +/- SEM). Confocal imaging, 63X objective (n = 3, maximum Z projection from approx. 30 zstacks). Scale bar:10 µm.

In HEK293T cells, NSP4 and NSP6 proteins formed inclusions/puncta mainly in the cytosol. The two proteins also significantly co-localised with reticular organisation of ER, labelled with calnexin (Pearson`s coefficient 0.62 ± 0.02 for NSP4 and 0.61 ± 0.04 for NSP6 respectively) (Fig. 4A, F). NSP7 exhibited a diffuse and essentially cytosolic presence, with no obvious co-localisation with the ER (Pearson coefficient 0.26 ± 0.05) (Fig. 4A, F). We observed no co-localisation between ORF3a and Golgi (130 kDa cis-Golgi matrix protein 1 (GM130)) (Pearson coefficient 0.30 ± 0.08) (Fig. 4B, F), or with autophagosomes (LC3 or Lamp1) (Pearson coefficient 0.23 ± 0.05 and 0.13 ± 0.01, respectively) (Fig. 4B, F). We observed a weak co-localization between ORF6 and calnexin, but no colocalization with LC3 and Lamp1 (Fig. 4C) (Pearson`s coefficient 0.55 ± 0.08; 0.35 ± 0.05 and 0.36 ± 0.07, respectively) (Fig. 4F). ORF7a mainly localized in the Golgi complex (Fig. 4D), but showed limited ER colocalization (Fig. 4D) (Pearson`s coefficient 0.50 ± 0.02; 0.38 ± 0.07, respectively) (Fig. 4F). ORF7b was weakly present at the ER with no significant co-localization with calnexin (Pearson`s coefficient 0.46 ± 0.07) (Fig. 4 D, F). ORF10 exhibited cytosolic distribution with no significant correlation with the ER (Pearson`s coefficient 0.36 ± 0.12), or with the nucleus (Fig. 4E, F). The membrane protein CoV M and the envelope protein CoV E were extensively localized in the ER (Fig. 4E) (Pearson`s coefficient 0.89 ± 0.03; 0.76 ± 0.06, respectively) (Fig. 4F).

### Subcellular distribution of SARS-CoV-2 proteins in BV2 cells

**Fig. 5:**
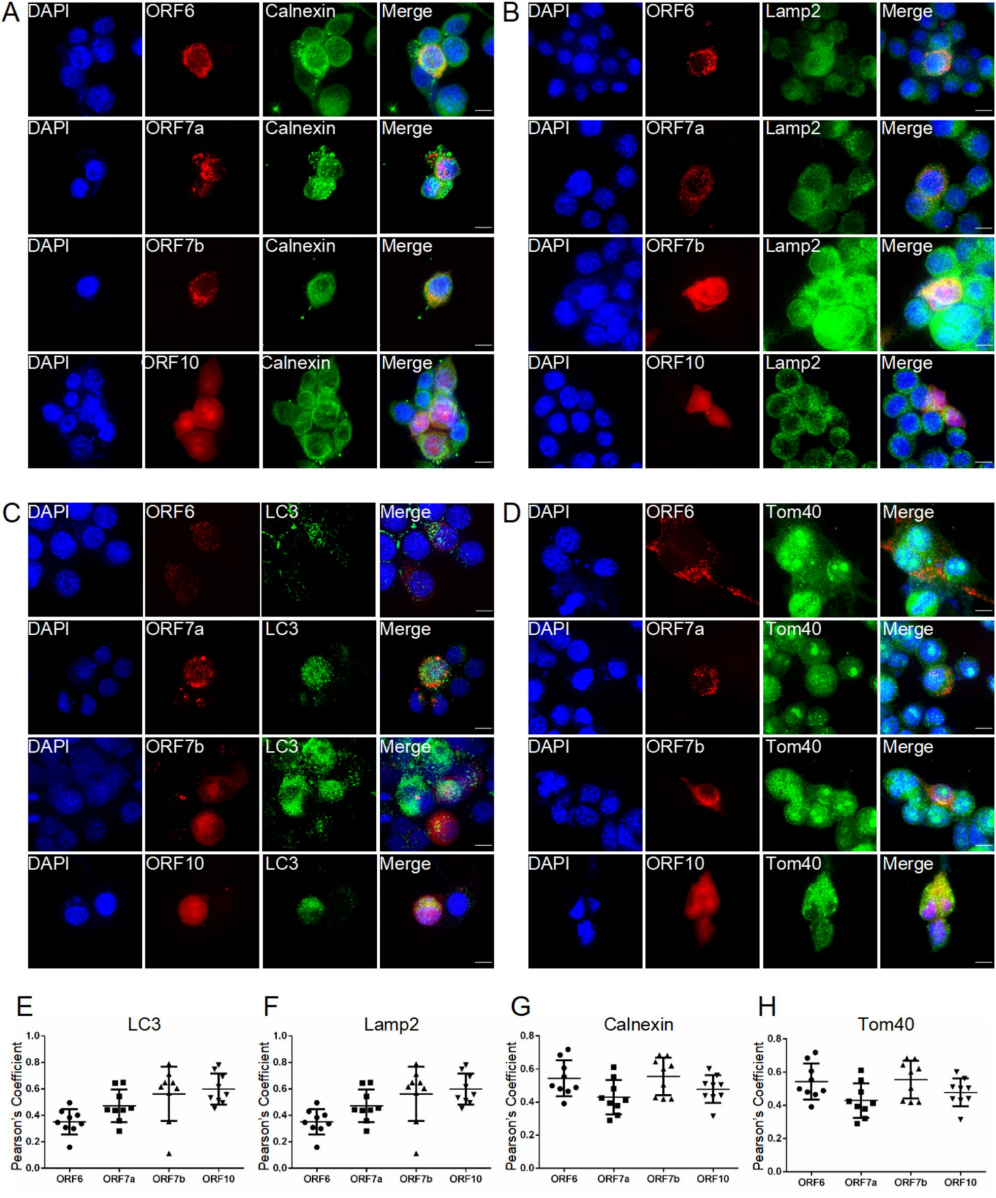
Subcellular localization of Sars-CoV-2 proteins in BV2 cells. Co-localization of Sars-CoV-2 proteins tagged with mCherry in electroporated BV2 cells with organelles markers: Calnexin (ER marker), translocase of outer mitochondrial membrane 40 (Tom40) (mitochondrial membrane marker), Lamp2 (lysosomal marker) and LC3 (autophagosome marker), 48 h after electroporation. A: ORF6, ORF7a, ORF7b and ORF10 (red) and ER (green); B: ORF6, ORF7a, ORF7b and ORF10 (red) and lysosome (green); C: ORF6, ORF7a, ORF7b and ORF10 (red) and autophagosome (green); D: ORF6, ORF7a, ORF7b and ORF10 (red) and mitochondria (green); E–H: colocalization quantification with analysis of Pearson`s coefficient for each Sars-CoV-2 protein with related organelle markers respectively. Statistical analysis (average +/SEM). Confocal imaging, 63X objective (n = 3, maximum Z projection from approx. 30 zstacks). Scale bar: 10 µm.

In BV2 cells, ORF6 formed cytosolic puncta and co-localized with ER (Pearson`s coefficient 0.54 ± 0.04) (Fig. 5A, G). No significant colocalization of ORF6 with autophagosomes (LC3), lysosomes (Lamp2) or mitochondria (Tom40) was observed (Pearson`s coefficient 0.35 ± 0.03, 0.33 ± 0.04, 0.19 ± 0.03 respectively) (Fig. 5B-D, E–H). ORF7a showed puncta-patterned distribution in both the cytoplasm and nucleus, with no significant colocalization in ER or autophagosomes (Pearson`s coefficient 0.43 ± 0.03, 0.47 ± 0.04, respectively) (Fig. 5A, C, E, G). The presence in lysosomes and mitochondria was weak (Pearson`s coefficient 0.37 ± 0.03, 0.30 ± 0.04, respectively) (Fig. 5B, D, F, H). We observed significant colocalization of ORF7b in autophagosomes, lysosomes and ER (Pearson`s coefficient 0.56 ± 0.07, 0.65 ± 0.04, 0.56 ± 0.04, respectively), and observed puncta in the cytosol (Fig. 5A-C, E–G). It showed minimal mitochondrial localization (Pearson`s coefficient 0.36 ± 0.03) (Fig. 5D, H). ORF10 showed diffused distribution in autophagosome and mitochondria (Pearson`s coefficient 0.60 ± 0.04, 0.55 ± 0.02, respectively), but not in lysosome or ER (Pearson`s coefficient 0.48 ± 0.04, 0.48 ± 0.03, respectively) (Fig. 5A-H). ORF10 was the one showing most significant colocalization with mitochondrial membrane (Fig. 5H), while ORF6 showed the strongest colocalization with ER (Fig. 5G) out of the four viral proteins investigated. ORF6 and ORF7b both colocalized with ER, and ORF7b colocalized with autophagy compartments (Fig. 5E-G).

### ORF6 alters ER and autophagy-lysosome function

**Fig. 6:**
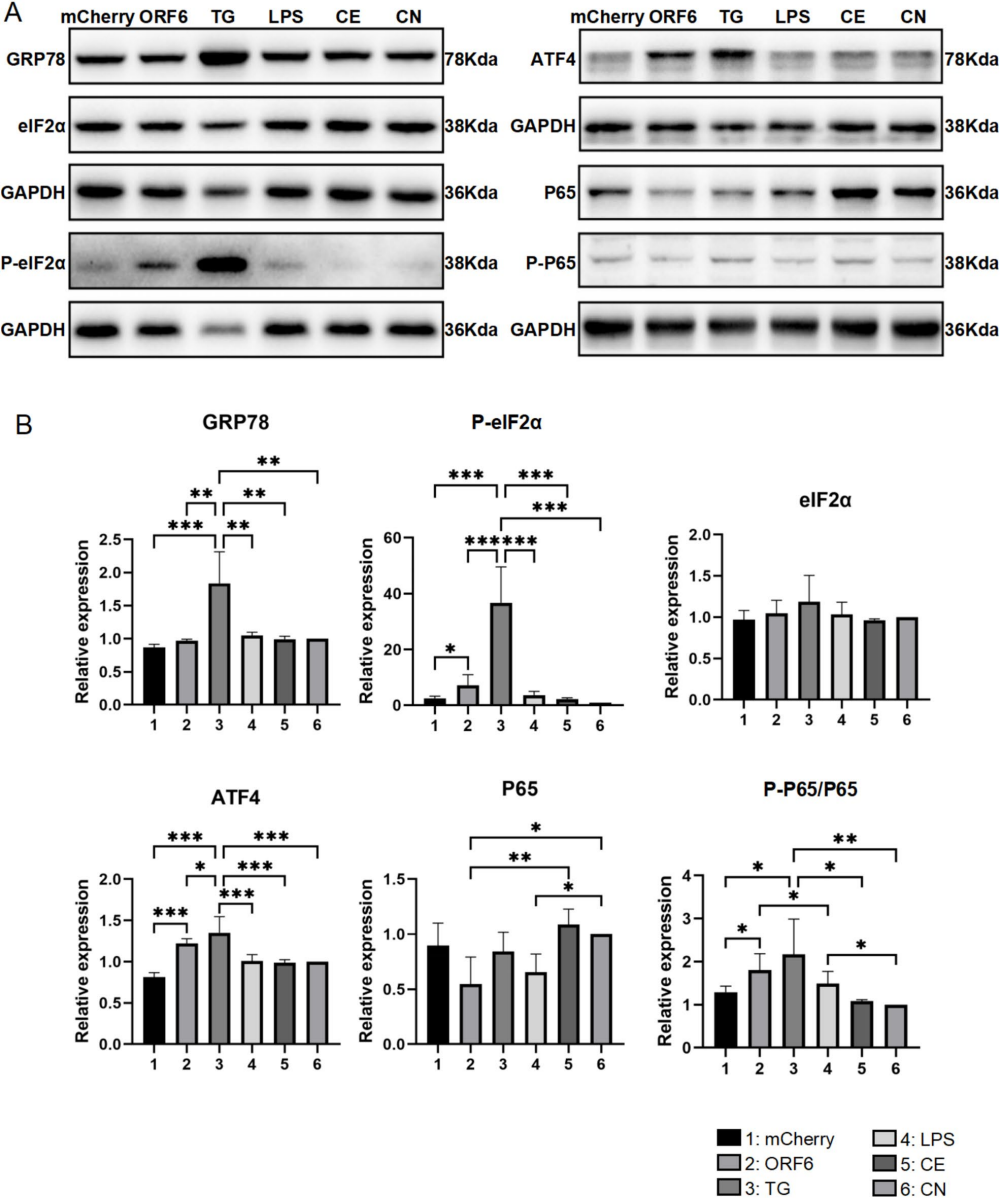
ORF6 induces ER stress. A: Western blot analysis of ER stress markers of GRP78, eIF2α, P-eIF2α, ATF4 and NF-κB marker of P65. B: Quantifications of the signal of the Western blots. CN, control group; CE control treated with electroporation. *p ≤ 0.05, **p ≤ 0.01 and ***p ≤ 0.001. The original bands for western blots shown in Fig. 6 are included in supplementary file “Western blot original data” file, as Blot 4–7.

Since ORF6 exhibited both proinflammatory effects and tendency to aggregate, we further detailed the cellular events underlying these effects. Based on the subcellular distribution of ORF6, we first studied ER stress-related proteins. In BV2 cells, the levels of glucose regulated protein 78 (GRP78), ER stress transducer protein eukaryotic translation initiation factor 2 (eIF2α) phosphorylation, and the downstream transcription inducer activating transcription factor 4 (ATF4) were evaluated^24^. The levels of ER stress were elevated, as indicated by the increased levels of eIF2α phosphorylation and the downstream ATF4 activation, without affecting GRP78 levels (Fig. 6). The ER stress-related NF-κB pathway was altered, as indicated by the phosphorylation of the transcription factor P65. The results suggest a proinflammatory pathway related to NF-κB signaling, which is mediated through induction of ER stress by ORF6, in agreement with the inflammasome-independent manner mentioned above.

**Fig. 7:**
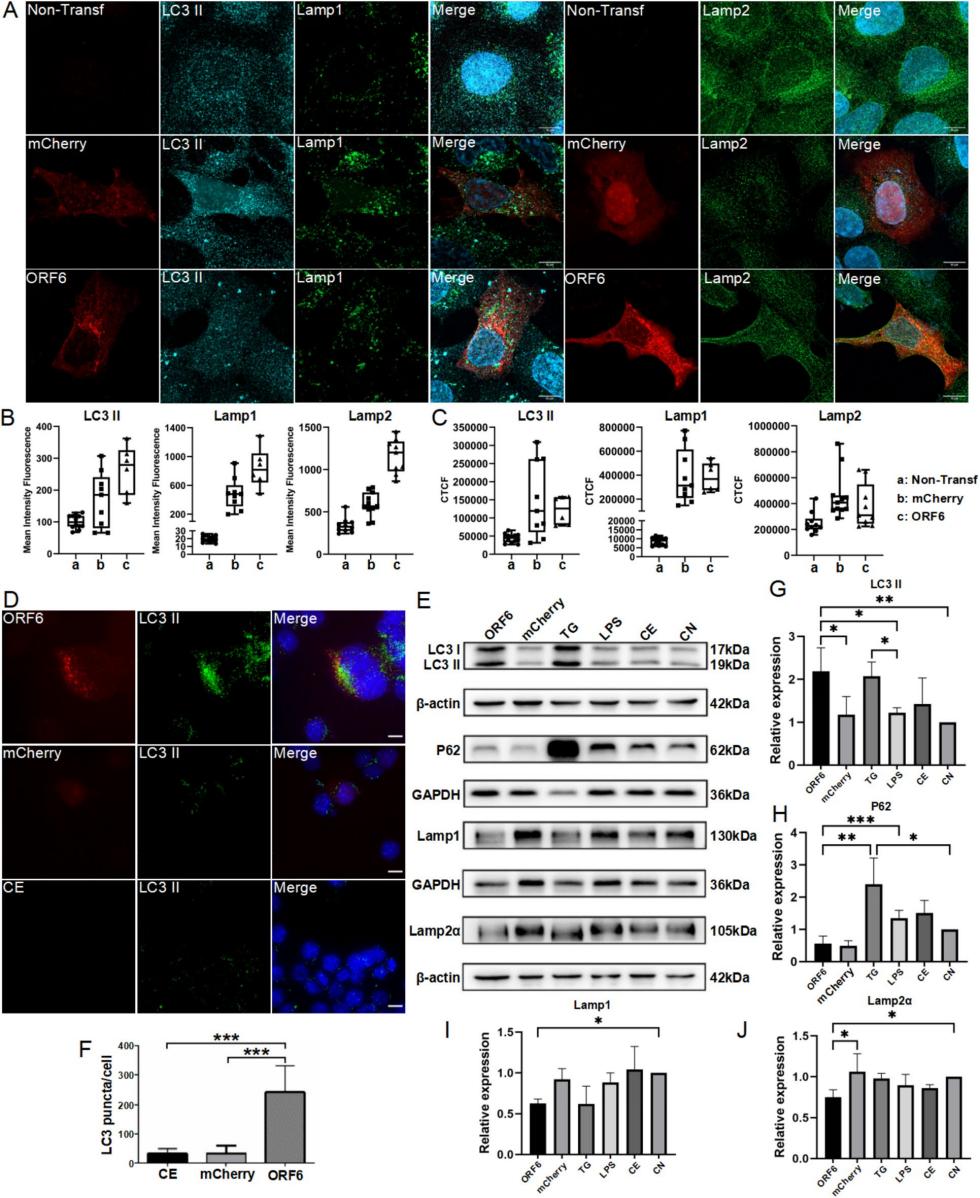
ORF6 induces autophagy-lysosome dysfunction. **A**: LC3B (cyan), Lamp1 (green) and Lamp2 (green) expression in HEK293T transfected with ORF6-mCherry. Individual cell ROI (**B**) were measured and Corrected Total cell Fluorescence (CTCF) (**C**) were calculated after subtractions of the background. **D**: BV2 cells were transfected with ORF6-mCherry showing expression of LC3B. **E**: Western blot of proteins on autophagy-lysosome pathway. **G**-**J**: quantification of protein expression levels of LC3, P62, Lamp1 and Lamp2α. F: LC3B positive signal quantification from D. Confocal imaging, 63X objective (n = 3, maximum Z projection from approx. 30 zstacks). CN, control group; CE control treated with electroporation; CE + LPS, control treated with electroporation and LPS treatment; ns, not significant; *p ≤ 0.05, **p ≤ 0.01 and ***p ≤ 0.001. Scale bar:10 µm. The original bands for western blots shown in Fig. 7 are included in supplementary file “Western blot original data” file, as Blot 1–3.

We next investigated the autophagy-lysosome pathway. Upon ORF6 transfection, autophagic and lysosomal functions were assessed by immunofluorescence in HEK293T cells (LC3 and Lamp1/2 markers, respectively). LC3 and Lamp1/2 mean fluorescence intensity was slightly increased in HEK293T cells after ORF6 transfection, relative to mCherry control, although not reaching statistical significance (Fig. 7A-C**,** with or without background subtraction (CTCF)). In BV2 cells, ORF6 accumulated in inclusions that partially colocalized with LC3 (Fig. 7D). Compared to control, the number of LC3 positive puncta was significantly increased, indicating an increase in autophagosome formation (Fig. 7F). In BV2 cells, we observed an increase of LC3 levels, and a decrease of Lamp and p62 proteins, compared to control, indicating the initiation of autophagy was not interrupted with the phagophore formation, but the autophagosome and autolysosome fusion were inhibited^25^ (Fig. 7E, G-J). In summary, a potential disruption of autophagy-lysosome function was suggested in HEK293T cells, while in BV2 the effects may be related to alterations of NF-κB signaling. The shown result link the HEK293T trends to the more definitive BV2 results, emphasizing the cell-type-specific responses.

### Torin1 treatment attenuates ORF6 aggregation

**Fig. 8:**
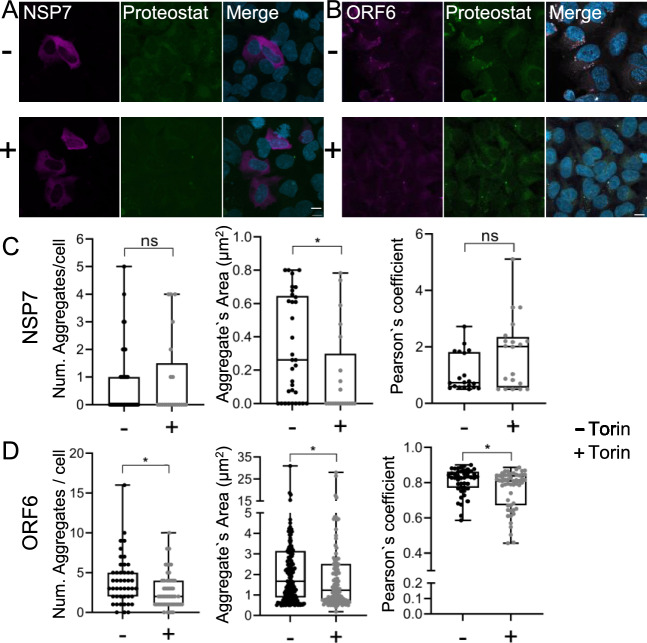
Induction of autophagy by Torin1 reduces the ORF6 protein aggregation. HEK293T cells were transfected with: NSP7 (**A**) and ORF6 (**B**) mcherry fused constructs for 24 h and treated with 1uM Torin1 for 1 h (+) and DMSO (-) only. C and D show colocalization of NSP7 and ORF6 with Proteostat dye and analysis per cell of: the number of aggregates, area of the aggregates and protein aggregation levels measured through Pearson`s coefficient. Confocal imaging, 63X objective (n = 3, maximum Z projection from approx. 30 zstacks). P < 0.05 (Mann Whitney test). Scale bar:10 µm.

In order to further investigate the effects of autophagy retardation induced by ORF6, we employed Torin 1, a synthetic mTOR inhibitor that blocks ATP-binding to mTOR and thus inactivates both mTORC1 and mTORC2, thereby may activating autophagy. HEK293T cells were transfected for 24 h and were then treated with 1 µM Torin1 for one hour. We then assessed protein aggregation by correlating mCherry puncta with Proteostat signal. Interestingly, we found that addition of Torin1 resulted in a statistically significant reduction of the number of inclusions and of the area of ORF6-mCherry inclusions per cell (Fig. 8B, D). No statistically significant changes were detected with NSP7 (Fig. 8A, C). For comparison, we also analysed the effect of Torin 1 on proteins forming small inclusions, such as ORF7a and ORF7b. We observed that the induction of autophagy by Torin1 significantly reduced the number of aggregates in ORF7a transfected cells (Supplementary Fig. 3).

## Discussion

The definition of long-COVID implies the existence of post-acute COVID-19 syndrome, which affects approximately one third of the patients, becoming an unneglectable problem facing the recovery from COVID-19 pandemic^26^. Neurological symptoms persistently affect a substantial number of patients in long COVID^27^, and a strong correlation between neurodegeneration and COVID-19 prognosis has been established. Epidemiologically, a fraction of hospitalized COVID-19 patients developed dementia and parkinsonism 6 months post infection^28^.

Several SARS-CoV-2 proteins—including the Spike protein, NSP6, NSP11, and ORF10—have been shown to form amyloid aggregates that exhibit neurotoxicity^18,29^. Computational studies further identified aggregation-prone regions in multiple viral proteins^19,22^, and cell-based experiments revealed that the Spike (S) and nucleocapsid (N) proteins can promote α-synuclein aggregation and toxicity^30^. These findings suggest a mechanistic link between SARS-CoV-2 protein aggregation and neurodegenerative proteinopathies. In this study, we demonstrate that selected SARS-CoV-2 proteins not only form intracellular inclusions but also trigger proinflammatory responses in microglia, mirroring pathological features of Parkinson’s disease (PD) and Alzheimer’s disease (AD)^31,32^.

As a single-stranded RNA virus, SARS-CoV-2 target essential cell organelles, whose dysfunction can facilitate pathways in favor of viral reproduction. Mitochondria are energy machinery of cells, meanwhile regulating turnover of lipids and process of apoptosis. It was reported that SARS-CoV-2 can target mitochondria and compete for the physiological energy production, meanwhile, promoting virus-mediated innate immune response^33^. Gordon et al. identified 332 high-confidence protein–protein interactions between SARS-CoV-2 and human proteins. Among these proteins, the CovM interacts with host mitochondrial proteins and facilitate cellular apoptosis^11^. Nevertheless, ORF8 and ORF6 have also been found affecting process of mitophagy and reactive oxygen species (ROS) generation^34,35^. Autophagy lysosome pathway is the main responder for harmful substances entering cellular environment, therefore SARS-CoV-2 proteins rely on the healthy status of lysosomes to be cleared out of cells. However, it was reported that ORFs, especially ORF7 can alter the autophagy lysosome degradation via changing the acidic pH, decreasing the number of acidic lysosomes^36^. Moreover, SARS-CoV-2 proteins such as CovE could decrease the calcium ion flow through ER and alter the pH within the Golgi apparatus and ER^37^. CovE was also shown changing the membrane permeability of ER and promoting IL-1β production, which in the end inducing neuroinflammation^38,39^. Therefore, in this study, we investigated the impact of viral proteins on three essential organelles including ER, mitochondria and lysosome. Other organelles such as autophagosome and Golgi apparatus are also influenced by SARS-CoV-2 proteins.

In our study, we demonstrated that certain viral proteins (ORF6, ORF7a, ORF7b and ORF10) induce microglia activation, possibly via the NF-κB signaling pathway, and involve an increase in the expression of TNF-α and IL-6, but no significant change in inflammasome related IL-1β. Interestingly, microglial NF-κB increase was shown to play a critical effect in the onset and pathology spreading in amyotrophic lateral sclerosis (ALS) and AD tauopathy^40,41^. Moreover, the increased secretion of TNF-α and IL-6 from microglia mimics the events happening in traumatic brain injury and metabolic disorders, which in turn increases the risk of neurodegeneration^42,43^. Therefore, it is important to consider that the effects we report on microglia may constitute another link between SARS-CoV-2 infection and neurodegeneration.

ORF6 was recently described to be localized at the ER and at a subset of the intracellular vesicles^44^. As summarized in Table 1, the SARS-CoV-2 protein inducing the strongest proinflammatory response is ORF6, and we found this protein to localize in the ER, suggesting a possible link between ER stress, aggregation, and neuroinflammation.

We also found that ORF7b and ORF10 show substantial localization with components of the autophagy-lysosome pathway. This similar subcellular distribution of ORF7a and ORF7b proteins was previously reported in Cos-7 cells^44^. Disruption of both ER and autophagy-lysosome function indicates the occurrence of alterations in proteostasis at the level of the unfolded protein response (UPR) and of protein clearance mechanisms^45,46^. These alterations may, in turn, contribute to additional protein aggregation, creating a vicious cycle that compromises cellular function. In total, our findings detail molecular alterations that can be used as putative targets for therapeutic intervention, with the goal of reducing secondary effects of COVID-19, including those related to neurological sequelae resulting from protein aggregation and neuroinflammation^47^.

As previously described, ORF3a expression caused endosomal morphology alteration^14,44^. Cells expressing ORF3a displayed enlarged early endosomes. ORF3a was previously described to exhibit a vesicular pattern, as it was found co-localised with late endocytic compartments and partially with GM130 (Golgi marker) as well as LC3 (autophagosome marker)^14^. However, with our HEK293T cells, we observed no significant presence of ORF3a at Golgi or autophagosome, possibly due to cell type difference.

It was reported that SARS-CoV-2 viral infection, even at mild stages, promoted the production of cytokines such as IL-6^48^. COVID-19 patient cerebrospinal fluid (CSF) sample presented neuroinflammatory profiles of elevated levels of IL-1β, TNF-α, IL-8, and IL-6^49,50^. Meanwhile, morphological and density alterations were found in patient brain microglia and astrocytes^51^. All these evidences suggest the neuroinflammatory inducing effect of SARS-CoV-2 proteins. As in our in vitro study, we observed in this study that SARS-CoV-2 proteins ORF6, ORF7a, ORF7b and ORF10 promote the BV2 microglia cell activation to a proinflammatory type, marked by increased mRNA levels of TNF-α and IL-6. Although, without the in-situ environment, the cytokine releasing phenotypes of virus promoted neuroinflammation was recapped. However, further in vivo examination of microglial activation and microvascular alterations will be needed for further confirmation of SARS-CoV-2 protein induced neuroinflammation.

While our study demonstrates that Torin 1 treatment reduces SARS-CoV-2 protein aggregation in HEK293T cells, several important limitations must be acknowledged. The aggregation-rescue effects were only shown in HEK293T cells, while the key pathological findings (ER stress, autophagy dysfunction) were primarily observed in BV2 microglial cells. We were unable to test Torin 1 in BV2 cells due to technical constraints of combining electroporation with prolonged drug treatment, creating a critical gap between mechanistic evidence and therapeutic potential. The 1-h Torin 1 treatment, while sufficient to observe acute effects on aggregation, may not reflect the sustained autophagy modulation needed for clinical relevance. Chronic treatment studies are warranted but were precluded by cell viability concerns. Although Torin 1 reduced ORF6 aggregation, we could not demonstrate parallel improvements in downstream pathologies (e.g., ER stress attenuation or cytokine reduction), leaving the functional significance of aggregate clearance unclear. As a potent mTORC1/2 inhibitor, Torin 1’s effects may extend beyond autophagy activation^52,53^. Our study did not include controls to distinguish autophagy-specific effects from other mTOR-related pathways. These limitations highlight the need for a better modeling system for studying the pro-inflammatory effect of SARS-CoV-2 proteins and application of targeted autophagy modulators to validate its therapeutic effects^51^. Future work should couple aggregation assays with functional readouts of proteostasis and inflammation.

Our study suggest that the key effects of SARS-CoV-2 proteins in BV2 cells were to induce inflammation dodging the inflammasome pathway. This forms a discrepancy between our findings and Albornoz et al., demonstrating SARS-CoV-2-induced inflammasome activation and IL-1β release in microglia^21^. The differences in results likely stem from distinct experimental models and mechanistic focuses. Albornoz et al. used full viral infection (SARS-CoV-2 particles) in human microglia, which engages multiple viral components and replication-dependent pathways, including the spike protein’s direct interaction with NLRP3. Our study examined individual viral proteins (ORF6, ORF7a, ORF7b, ORF10) expressed in BV2 cells, isolating their effects without viral replication. This approach may not recapitulate the combinatorial inflammatory triggers of intact virions. The spike protein (S) in Albornoz et al. was shown to activate NF-κB and NLRP3, driving IL-1β release^54^. In contrast, our tested proteins (ORF6/7a/7b/10) induced NF-κB-mediated TNF-α/IL-6 upregulation without IL-1β elevation, suggesting inflammasome-independent pathways. This aligns with ORF6’s reported role in ER stress and autophagy disruption, which may favor NF-κB over NLRP3 activation. BV2 cells, while widely used, may not fully mirror primary human microglia responses. Differences in TLR/NLRP3 expression or signaling could explain the lack of IL-1β induction. Our 48-h post-transfection timepoint (to allow cell recovery post-electroporation) might miss earlier inflammasome priming events. The absence of secondary signals (e.g., ATP) required for NLRP3 activation in our system could also contribute. These differences highlight the complexity of SARS-CoV-2 neuroinflammation, where whole-virus infection and individual protein effects may engage divergent pathways. We have clarified this distinction in the revised Discussion, citing Albornoz et al. to contextualize our findings within broader literature. Future studies comparing full viral infection versus individual protein expression in primary microglia could resolve these mechanistic nuances.

## Conclusions

In summary, our study investigated the aggregation propensity, subcellular distribution, and pro-inflammatory effects of selected SARS-CoV-2 proteins, revealing that proteostatic dys-homeostasis and neuroinflammation might offer novel targets for intervention for combating the neurotoxicity caused by SARS-CoV-2 infection.

## Materials and Methods

### Cell lines and plasmids

In this study, we used human embryonic kidney cells HEK293T and mouse microglia cells–BV2, purchased from ATCC^@^. Mammalian expression constructs for NSP4-mCherry, NSP6-mCherry, ORF3a-mCherry, NSP7-mCherry and EGPF-CoV E were generously gifted by Miserey-Lenkei *et al*^55^ (Addgene plasmid #165,132, #165,133, #165,134, and #165,123 respectively). ORF6, ORF7a, ORF7b, ORF10 and CoV M CDS sequence from SARS-CoV-2 reference genome (Wuhan/Hu-1/2019) were flanked by the addition of the restriction enzymes sequence (NheI and XhoI) and synthetized using geneart service (ThermoFisher, Darmstadt, Germany). After linearization of the constructs with the restriction enzymes digestion, SARS-CoV-2 CDS sequences were assembled into the mCherry N1 expression plasmid (Takara, Saint-Germain-en-Laye, France) using NEBuilder HiFi DNA Assembly Cloning Kit (New England BioLabs, Frankfurter, Germany). All resulting constructs were verified by DNA sequencing. mCherry fluorescence was used for tracking the subcellular localization of the viral proteins.

### Cell culture, transfection and treatment

The HEK293T cells were grown in Dulbecco’s modified Eagle medium (DMEM), supplemented with 10% fetal bovine serum (FBS) and 1% penicillin and streptomycin (P/S). Mouse microglia cell line BV2 were grown in DMEM^@^GlutaMAX (Gibco), supplemented with 10% FBS (ThermoFisher, Darmstadt, Germany) and 1% P/S.

Transfection of HEK293T was performed using Metafectene Pro reagents (Biontex, Munich, Germany) and following manufacturer protocol. Briefly, HEK293T cells passage 10–12 were cultured 24 h prior to transfection. 5 × 10^5^cells/well were seeded with 1 ml DMEM + 10% FBS without 1% P/S. Cell confluence at transfection was 80–90%. Ratio of 1:3 (0.5 µg DNA: 1.5 µl Metafectene-PRO) was optimized in order to reach the highest transfection efficiency with only very moderate cell death. Plasmid DNA was complexed with 50 µl of OPTI-MEM in one tube and Metafectene-PRO was complexed with 50 µl of OPTI-MEM in another tube. Solutions were left for 5 min, after which they were combined and left for another 20 min at room temperature. Transfection mixture was added dropwise to the cells and left for 24 h in the incubator (37ºC, 5% CO_2_).

Transfection of BV2 was performed with Gene Pulser Xcell Electroporation Systems (Bio-Rad). 1 × 10^5^ BV2 cells were transferred into electroporation cuvette (Gene Pulser/MicroPulser Electroporation Cuvettes, 0.1 cm gap #1,652,089), with plasmids inoculated under 70 V. After 4 h of electroporation, the culture media of BV2 was exchanged to culture medium. The current data interpretation has been carefully cross-validated with untransfected controls.

Torin 1 (inh-Tor-1, InvivoGen, Toulouse, France) was dissolved in dimethyl sulfoxide (DMSO; Sigma, Taufkirchen, Germany) and added to the cell culture medium 24 h post-transfection at a final concentration of 1 µM and incubated for 1 h at 37 °C and 5% CO_2_.

### Immunofluorescence and confocal imaging

HEK293T and BV2 cells were grown on coverslips (13 mm diameter) in 24-well plates and transiently transfected using the different SARS-CoV-2 constructs. With BV2 cells, normalization experiments were performed to ensure that similar concentration of plasmids were introduced into the cells, as the transfection efficiency was comparatively low, unlike HEK cells. 24 and 48 h post transection, the cells were fixed using 4% paraformaldehyde (PFA 4%, Roth) for 15 min at room temperature, and permeabilized with PBS containing 0.5% Triton X-100 for 20 min at room temperature. After blocked in 3% bovine serum albumin (BSA) in PBS for 1 h at room temperature, primary antibody (Table 2) incubation was carried out overnight at 4 °C, followed by secondary antibody incubation for 1 h at room temperature and DAPI to stain the nuclei. The coverslips were mounted on microscope slides using Mowiol mounting medium (10% (w/v) Mowiol 4–88, 25% (w/v) Glycerol, 25% (v/v) water, 50% (v/v) Tris–Cl 0.2 M pH 8.5, 2.5% (w/v) DABCO). Images were acquired using Zeiss LSM900 and Nikon Eclipse Ti2, analyzed with Fiji ImageJ and NIS-Elements AR 5.21.00.

**Table 2:** Antibodies used for immunofluorescence.

Primary antibodies	Vendor	CatLog number	Species	Dilution
GM130	BD Biosciences	610,822	Mouse	1:500
Lamp1	Abcam	ab62562	Rabbit	1:500
LC3	MBL	PM036	Rabbit	1:500
Calnexin	Abcam	ab22595	Rabbit	1:500
HSP 60	Santa Cruz	sc-13966	Rabbit	1:500
LC3B	Cell Signaling Technology (CST)	3868S	Rabbit	1:500
GAPDH	Proteintech	10,494–1-AP	Rabbit	1:2000
β-actin	Abcam	ab8227	Rabbit	1:2000
GRP78	Abcam	ab21685	Rabbit	1:1000
eiF2α	CST	9722	Rabbit	1:1000
P-eiF2α	CST	9721S	Rabbit	1:1000
ATF4	CST	11,815	Rabbit	1:1000
P65	Santa Cruz	sc-8008	Mouse	1:1000
P-P65	ZENBIO	R380738	Rabbit	1:500
LC3	Abcam	ab48394	Rabbit	1:1000
P62	Abcam	ab109012	Rabbit	1:1000
Lamp1	Abcam	ab24170	Rabbit	1:1000
Lamp2α	Abcam	ab18528	Rabbit	1:1000
**Secondary antibodies**	**Vendor**	**CatLog number**	**Species**	**Dilution**
Alexa Fluor 488	Invitrogen	A11008	goat anti-rabbit	1:1000
Alexa Fluor 488	Invitrogen	A11029	goat anti-mouse	1:1000
Alexa Fluor 680	Invitrogen	A10043	donkey anti-rabbit	1:1000
Alexa Fluor 488	Abcam	ab150077	goat anti-rabbit	1:1000
HRP conjugated	Abcam	ab205719	goat anti-mouse	1:1000
HRP conjugated	CST	7074	goat anti-rabbit	1:1000

### Protein aggregation assay

The aggregation status of the SARS-CoV-2 proteins was monitored by ProteoStat Protein Aggregation Assay (Enzo Life Sciences, Farmingdale, NY, USA) according to the manufacturer’s protocol. Briefly, 24 h after HEK293T cells transfection with the SARS-CoV-2 constructs, cells were fixed with 4% PFA. ProteoStat detection dye was added for 30 min in the dark at room temperature. The fluorescent signal was measured by confocal microscopy using the excitation at 488 nm and emission at 575 nm after linear Un-mixing for the final image (Z-stack) with the resolution accordingly to the best spectra separation settings between Proteostat dye and mCherry.

### Measurements of cytokine release

To detect the expression level of pro-inflammatory cytokines induced by SARS-CoV-2 proteins in BV2, total RNA from SARS-CoV-2 proteins transfected BV2 cells was isolated using TRIzol (Thermofisher, Waltham, USA). Reverse transcription of total RNA to complementary DNA using commercial kits (Takara). Quantitative polymerase chain reaction (qPCR) programs were designed as follows: 30 s at 95 °C, followed by 5 s at 95 °C and 30 s with 60 °C for 40 cycles. Relative mRNA expression levels were quantified using the 2^–ΔΔCt^ method and normalized to glyceraldehyde-3-phosphate dehydrogenase (GAPDH) as the housekeeping gene.

### Western blot analyses

To determine the expression levels of proteins involved in the autophagy lysosome pathway, BV2 cells were homogenized in radioimmunoprecipitation assay buffer containing 1% protease (Bimake, Houston, USA) and 1% phosphatase (CWBIO, Beijing, China) inhibitors. The homogenates were centrifuged at 4 °C, 13 000 rpm for 30 min, and the protein concentrations in the supernatant were quantified using a bicinchoninic acid kit (Beyotime, Shanghai, China). Protein solutions were mixed with a 5 × loading buffer and denatured in a water bath at 95 °C for 10 min. Approximately 20 µg of each protein sample were loaded and separated by 12% sodium dodecyl sulphate–polyacrylamide gel electrophoresis (Solarbio, Beijing, China), followed by transfer onto polyvinylidene fluoride membranes (Millipore, Billerica, USA). After blocking (5% skim milk in Tris-buffered saline with 0.1% Tween-20) at room temperature for 1 h, the membranes were incubated with primary antibodies at 4 °C overnight, followed by incubation with horseradish peroxidase-conjugated secondary antibodies (Table 2) at room temperature for 1 h. In the end, the protein bands were developed using enhanced chemiluminescence. The band intensities were analysed using ImageJ software, with normalization to the levels of GAPDH and β-actin.

### Imaging analyses

Images were acquired using a Zeiss LSM 900 confocal microscope with a 63 × oil immersion objective. mCherry and ProteoStat signals were captured using dedicated laser lines and filter sets to prevent channel bleed-through. For each condition, single Z-stack images were acquired with consistent settings. Maximum intensity Z-projection images were generated from approximately 30 Z-stack frames across all experimental conditions. The ProteoStat® Aggresome dye was excited with a 488 nm laser, and spectral unmixing protocols were applied to resolve its emission from mCherry fluorescence. Analysis was performed using Fiji/ImageJ (version [insert]) with the JACoP (Just Another Colocalization Plugin) and built-in tools. Thresholding: Raw images were split into individual channels. The mCherry (ORF6-mCherry) and ProteoStat channels were independently thresholded using the Costs method to segment fluorescent signals. Colocalization Analysis: JACoP was used to calculate Manders’ overlap coefficients (M1, M2) and Pearson’s correlation coefficients from thresholded images, assessing colocalization between SARS-CoV-2 proteins, subcellular organelles, and ProteoStat staining. Line-scan profiles across transfected cells further evaluated aggregation states. Aggregate Quantification: The“Analyze Particles”function quantified mCherry-positive aggregates per cell, measuring particle count and total area. Regions of interest (ROIs) were manually defined using DAPI and mCherry channels as reference. Protein Level Measurement: Corrected total cell fluorescence (CTCF) was calculated for LC3B and Lamp2 levels, comparing SARS-CoV-2 proteins to controls within individual cell ROIs.

Data from ≥ 15 cells per condition across 3 independent experiments were analyzed in GraphPad Prism (version 8). Torin1-treated vs. untreated groups were compared using the non-parametric Mann–Whitney U test (*p* < 0.05 considered significant).

### Statistical analyses

Statistical analysis was conducted using GraphPad Prism 8 software (GraphPad Software, Inc.). The Shapiro–Wilk normality test was applied to test data distribution. Under a normal distribution, variables were compared using the two-tailed unpaired t-test, while under asymmetrical distribution variables were analysed using the non-parametric two-tailed Mann–Whitney test. The level of statistical significance was set as *p* ≤ 0.05. Co-localization analysis were performed using ImageJ JaCOP plugin, Pearson`s ecoefficiency was assessed with 3 independent z-stacks and individual cell region of interest (ROI) on maximum Z intensity projection images. Values represented: average +/- SEM. With Pearson’s Coefficient analyses, values larger than 0.5 were designated for positive colocalization, while values lower than 0.5 indicated no significant colocalization. In western blot experiments, band intensities were quantified using ImageJ. Data were normalized to loading controls. One-way ANOVA with Tukey’s post-hoc test was used for multiple comparisons. p-values are now accompanied by the specific test used in all figure legends.

## Supplementary Information

Supplementary Information 1.

Supplementary Information 2.

## Data Availability

All data generated during this study are included in the manuscript, figures and supplementary information. All data will be available from the corresponding author upon reasonable request.

## Abbreviations

AD: Alzheimer’s disease
ALS: Amyotrophic lateral sclerosis
APR: Aggregation-prone regions
ATF4: Activating transcription factor 4
BSA: Bovine serum albumin
CNS: Central nervous system
COVID-19: Coronavirus disease 2019
CTCF: Corrected total cell fluorescence
DMEM: Dulbecco’s modified Eagle medium
E proteins: Envelope proteins
ER: Endoplasmic reticulum
eIF2α: Eukaryotic translation initiation factor 2FBS: Fetal bovine serum
GAPDH: Glyceraldehyde-3-phosphate dehydrogenase
GM130: 130 KDa cis-Golgi matrix protein 1
GRP78: Glucose regulated protein 78
HEK: Human embryonic kidney cell line
Lamp2: Lysosome-associated membrane glycoprotein 2
LC3: Microtubule-associated protein 1A/1B-light chain 3
M proteins: Membrane proteins
NSPs: Nonstructural proteins
N proteins: Nucleocapsid proteins
PFA: Paraformaldehyde
P/S: Penicillin and streptomycin
PD: Parkinson’s disease
qPCR: Quantitative polymerase chain reaction
ROI: Region of interest
SARS-CoV-2: Severe acute respiratory syndrome coronavirus 2
S proteins: Spike proteins
Tom40: Translocase of outer mitochondrial membrane 40
UPR: Unfolded protein response
