# Supplementary material for: Aggregation potency and proinflammatory effects of SARS-CoV-2 proteins
Source: Sci Rep. 2025 Aug 4;15:28446. doi: 10.1038/s41598-025-10013-1 (PMC12321994; doi:10.1038/s41598-025-10013-1)
Supplement: Supplementary file 1 — Supplementary Information 1. [file 41598_2025_10013_MOESM1_ESM.docx]

**Supplementary figures**

**
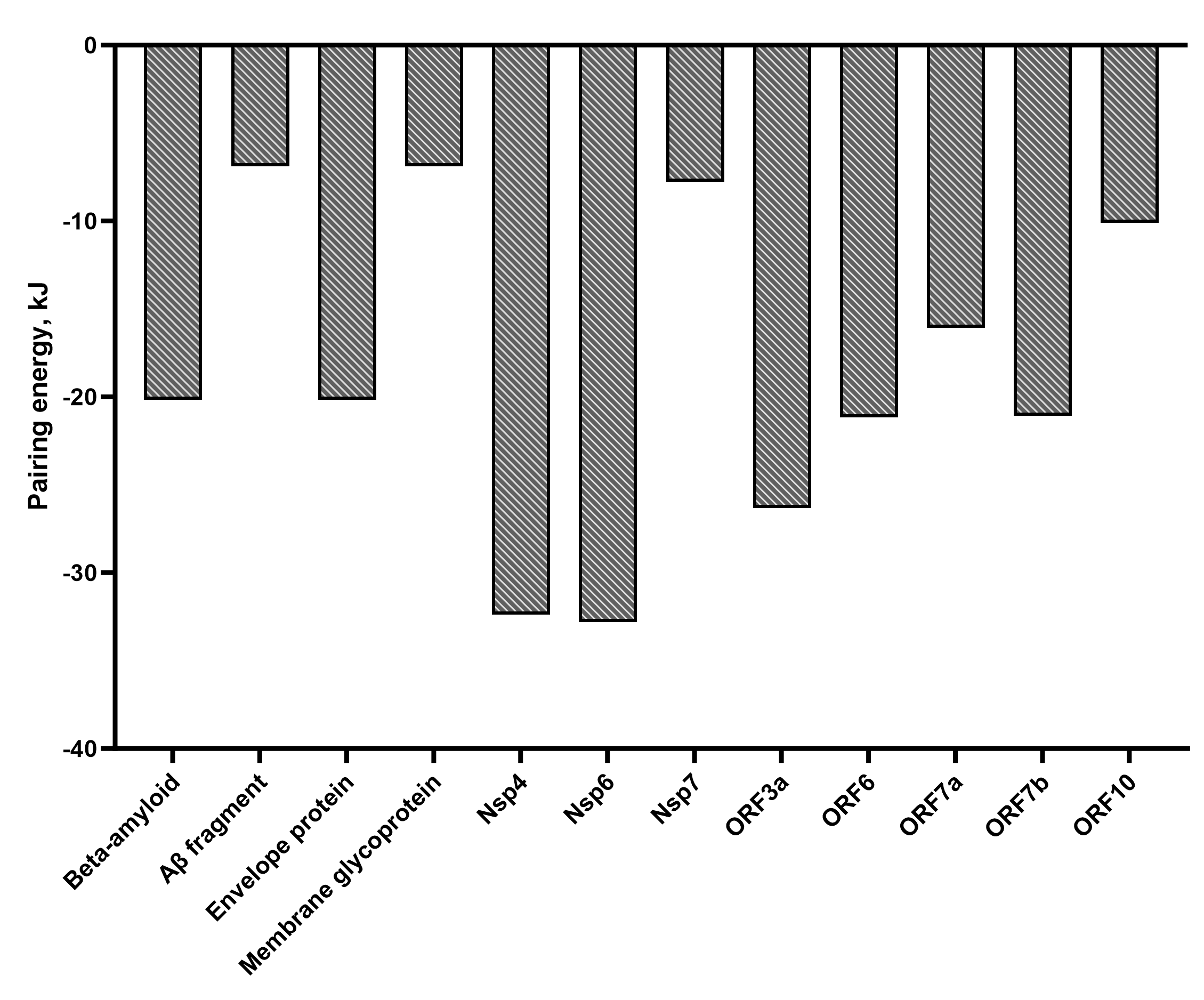
**

**Supplementary figure 1.** Prediction of aggregation propensities of SARS-CoV-2 proteins. The graph shows the predicted aggregation potency of SARS-CoV-2 proteins using PASTA2.0 algorithm. Beta-amyloid and Aβ fragments are applied as positive controls, along with SARS-CoV-2 protein with high aggregation propensity. The PASTA2.0 algorithm was used with default parameters to predict the aggregation propensity of SARS-CoV-2 proteins. Specifically: energy threshold for aggregation-prone regions (APRs): ≤ -5.0 kcal/mol. Sequences with pairing energies below this threshold were considered aggregation-prone. Window size: 5 amino acids (default), sliding across the entire protein sequence. Output: Pairing energies for all possible sequence segments, with lower (more negative) values indicating higher aggregation propensity.

**
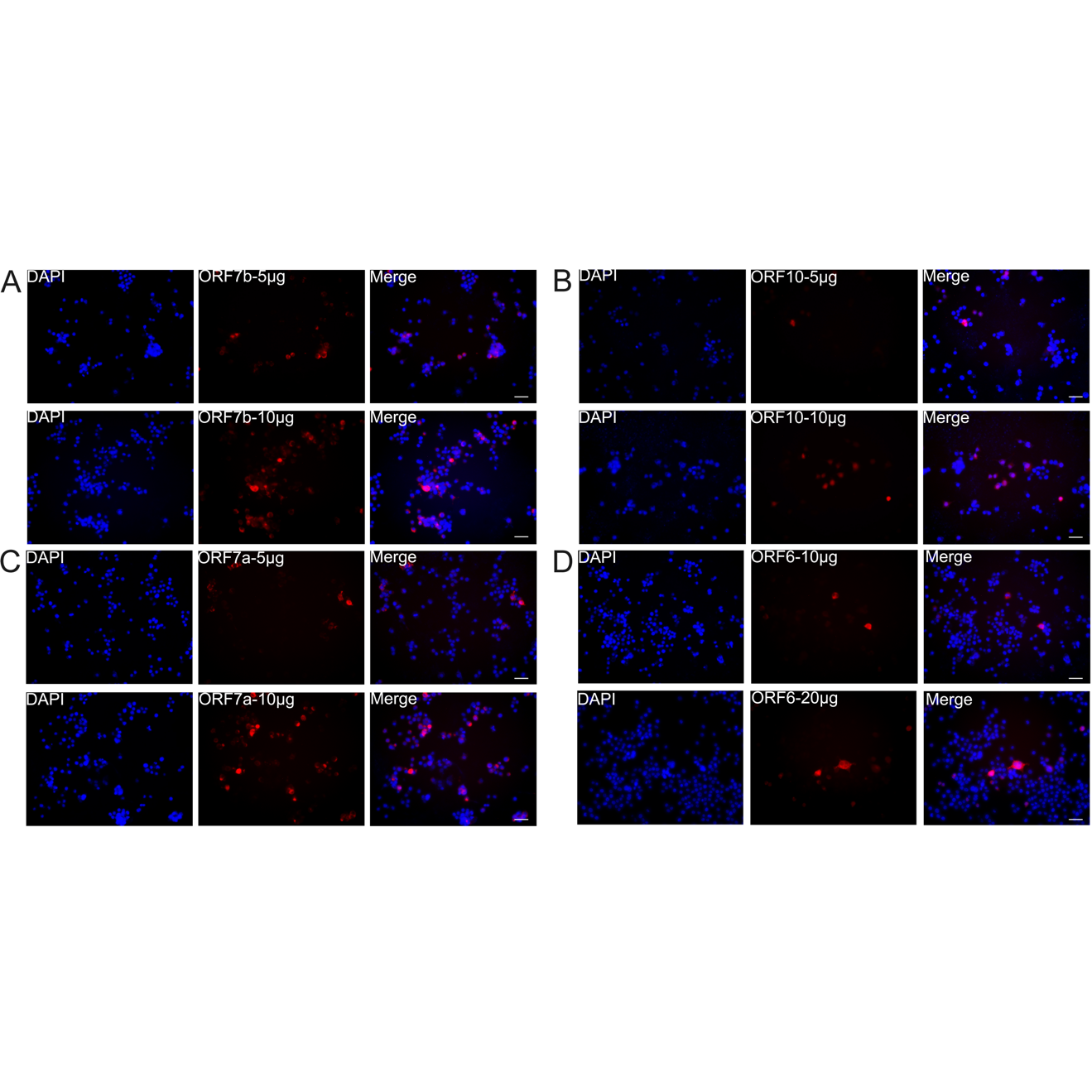
**

**Supplementary figure 2.** Expression of Sars-CoV-2 proteins in BV2 cells 48 hours after electroporation. Images showing expression of ORF6 (D), ORF10 (B), ORF7a (C) and ORF7b (A) in BV2 cells, with nucleus staining. Electroporation of NSP4, NSP6, NSP7, ORF3a and CoV M were also performed, without visible entry of fused fluorescent signals. Scale bar: 50µm.

**Supplementary figure 3.** Induction of autophagy alters the aggregation pattern of ORF7a and ORF7b. HEK293 cells were transfected with: ORF7a (A) and ORF7b (B) mcherry fused constructs for 24h and treated with 1uM Torin1 for 1h (+) and DMSO (-) only. C and D indicate colocalization of ORF7a and ORF7b, respectively, with Proteostat dye and analysis per cell of: number of aggregates, area of the aggregates and protein aggregation levels measured through Pearson`s coefficient. Confocal imaging, 63X objective (n=3, maximum Z projection from aprox. 30 zstacks ). p<0.05 (Mann Whitney test). Scale bar:10µm. ORF7a and ORF7b represent constructs that were shown to induce small dot-like and puncta of big aggregates. Treatment with Torin 1 significantly reduces the number of ORF7a aggregates per cell but not the area or the overall aggregation levels of this protein within the cell.
