# Supplementary material for: Aggregation potency and proinflammatory effects of SARS-CoV-2 proteins
Source: Sci Rep. 2025 Aug 4;15:28446. doi: 10.1038/s41598-025-10013-1 (PMC12321994; doi:10.1038/s41598-025-10013-1)

**Western blots original data**


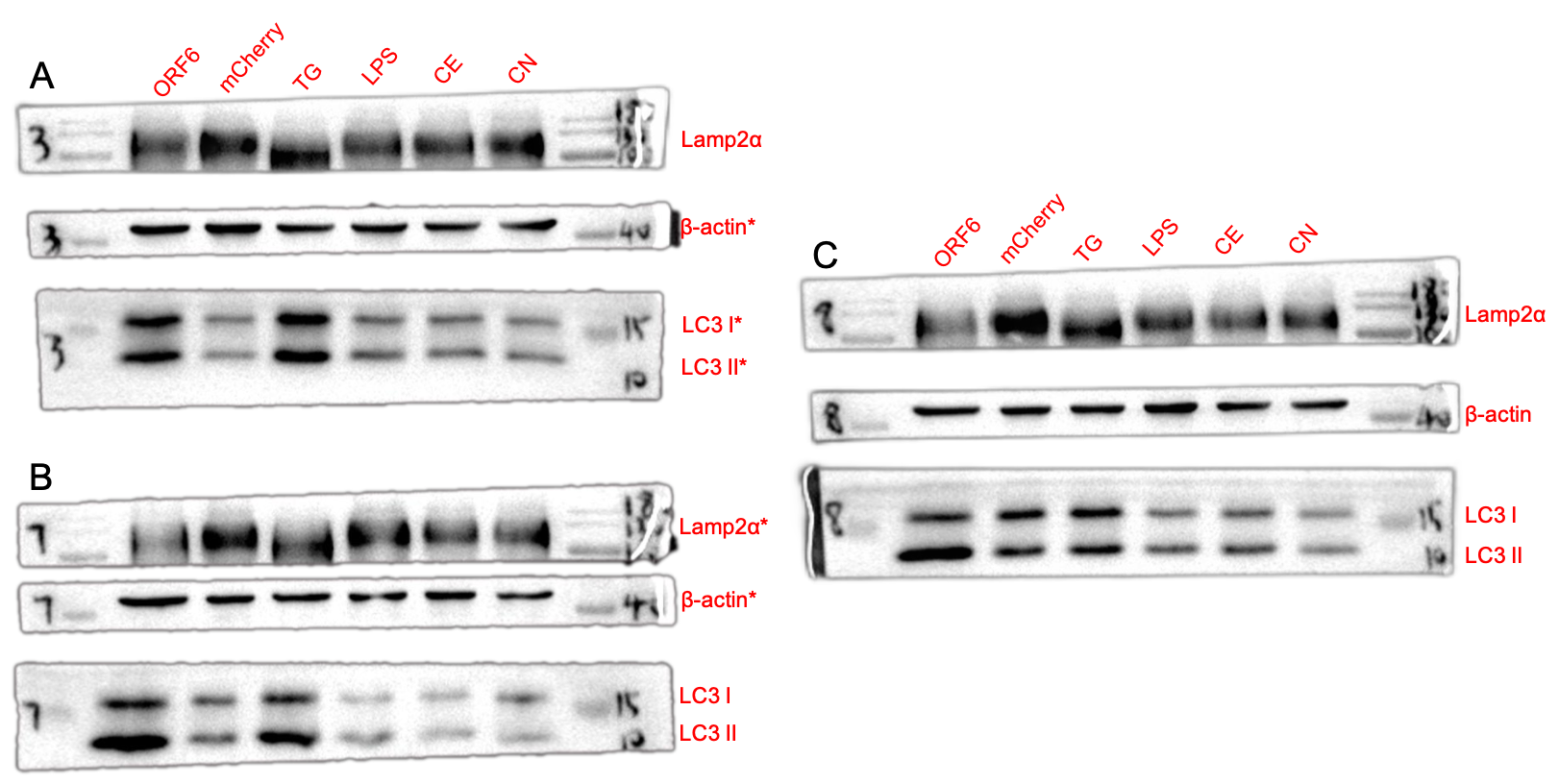
**Blot 1:** the western blot was repeated three times (A-C) for quantification. The bands of LC3 I and II, β-actin and Lamp2α in figure 7 are indicated in A, B. (**_*_**) indicates representative bands shown in figure 7.

**Blot 2:** the western blot was repeated three times (A-C) for quantification. The bands of P62 and GAPDH in figure 7 are indicated in A. (**_*_**) indicates representative bands shown in figure 7.


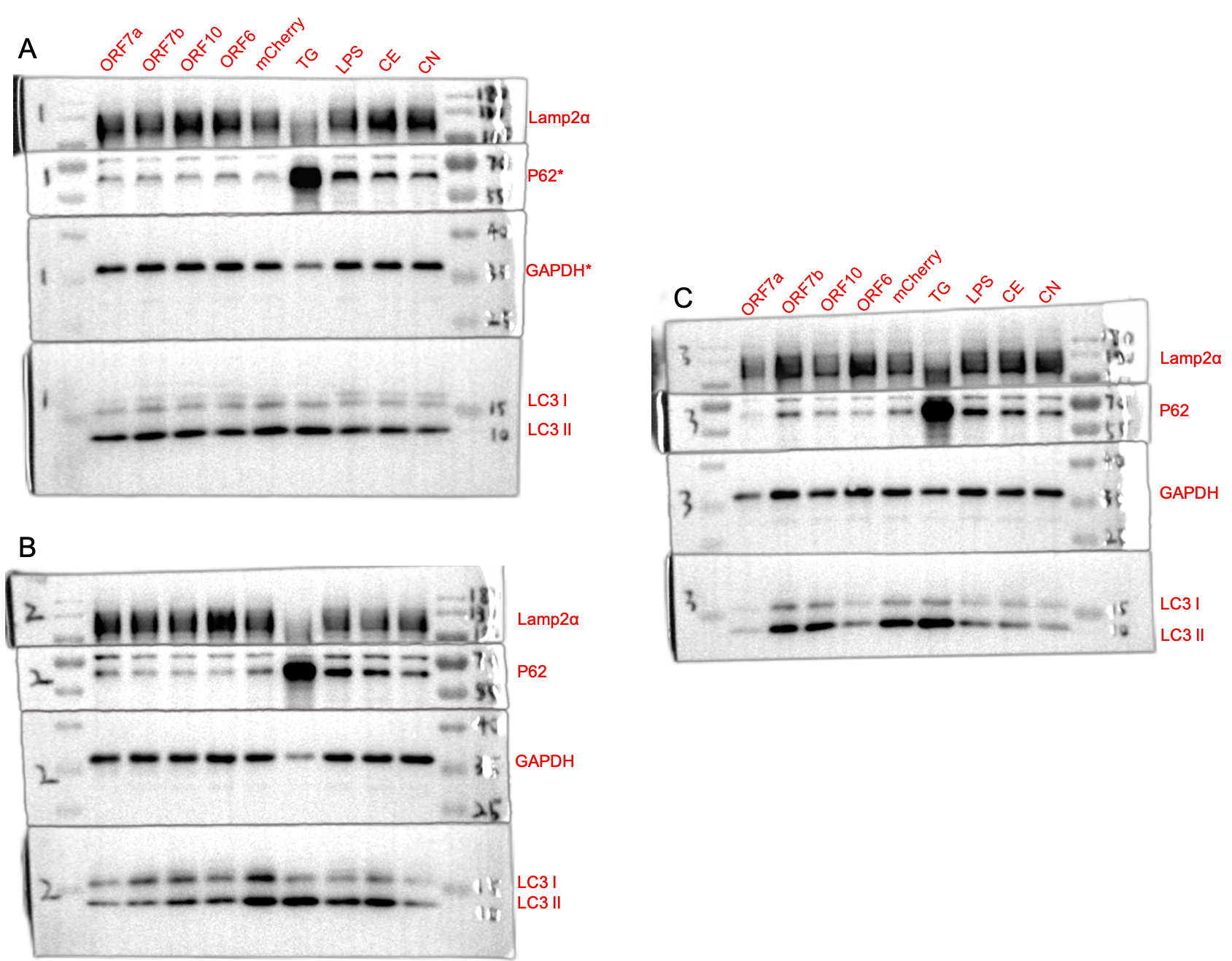


**
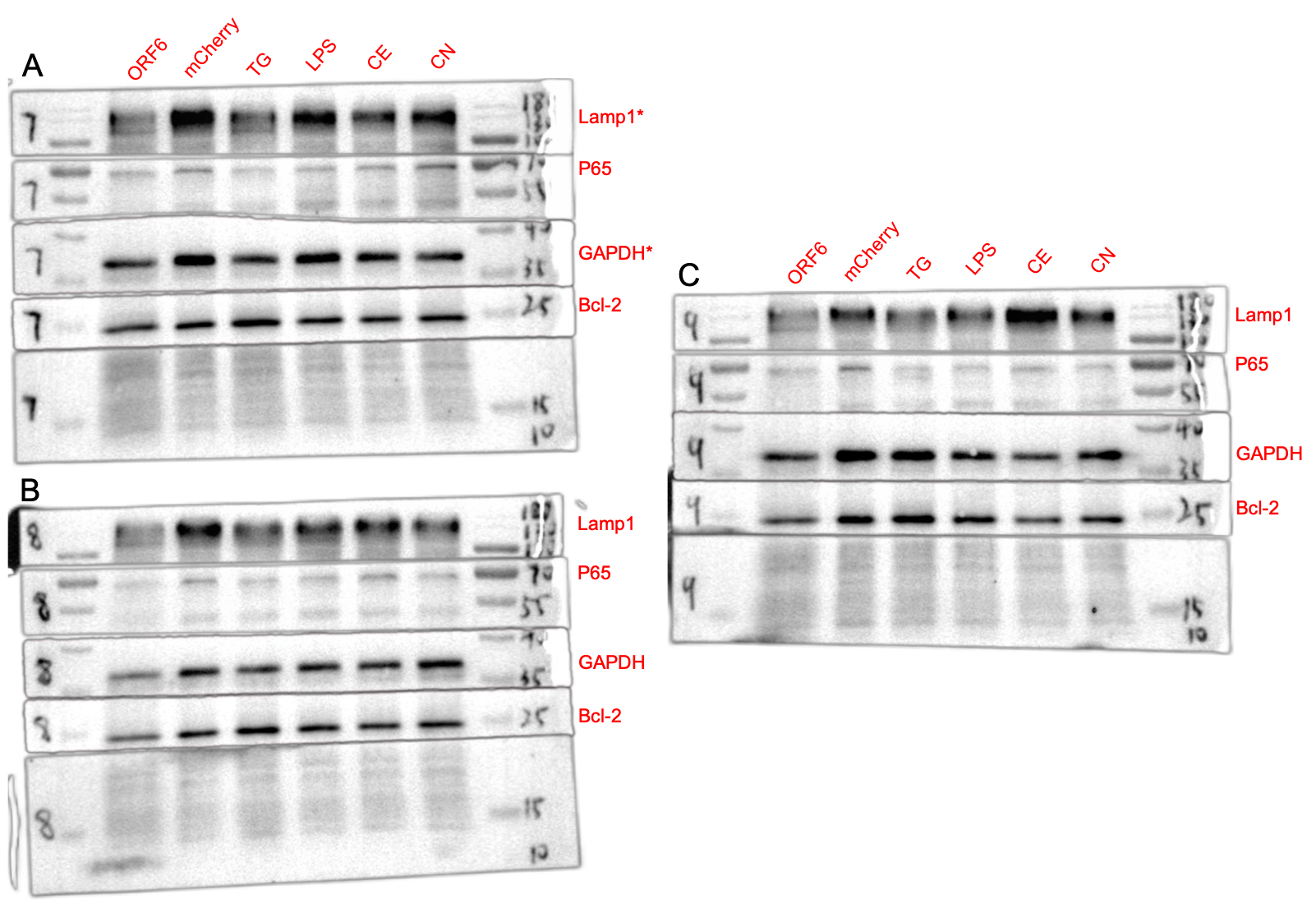
Blot 3:** the western blot was repeated three times (A-C) for quantification. The bands of Lamp1 and GAPDH in figure 7 are indicated in A. (**_*_**) indicates representative bands shown in figure 7.

**Blot 4:** the western blot was repeated three times (A-C) for quantification. The bands of GRP78, eIF2α and GAPDH in figure 6 are indicated in A and D. (**_*_**) indicates representative bands shown in figure 6. Due to the similar size of GAPDH and eIF2α, the membrane was stripped and re-incubate for eIF2α after GAPDH blotting.


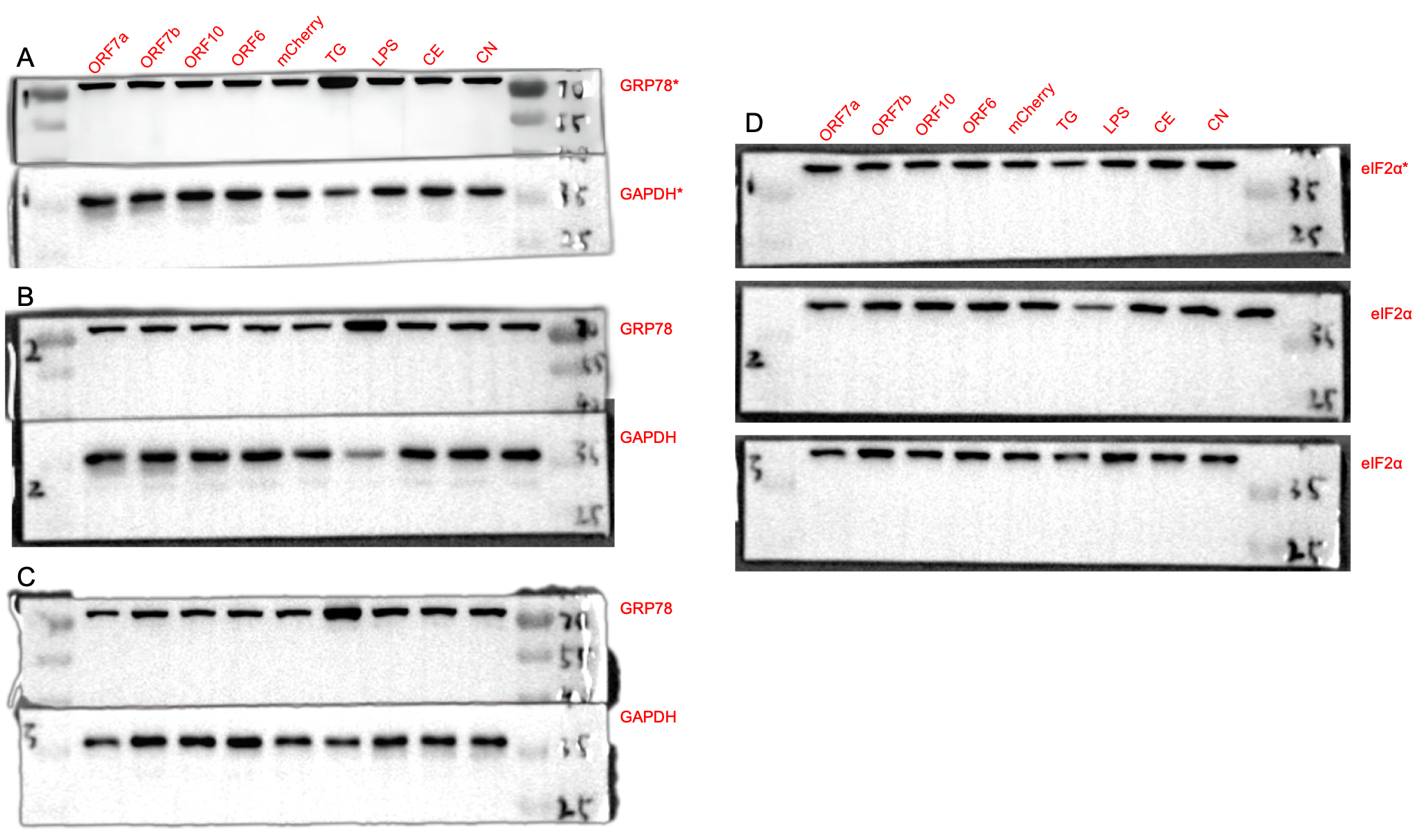


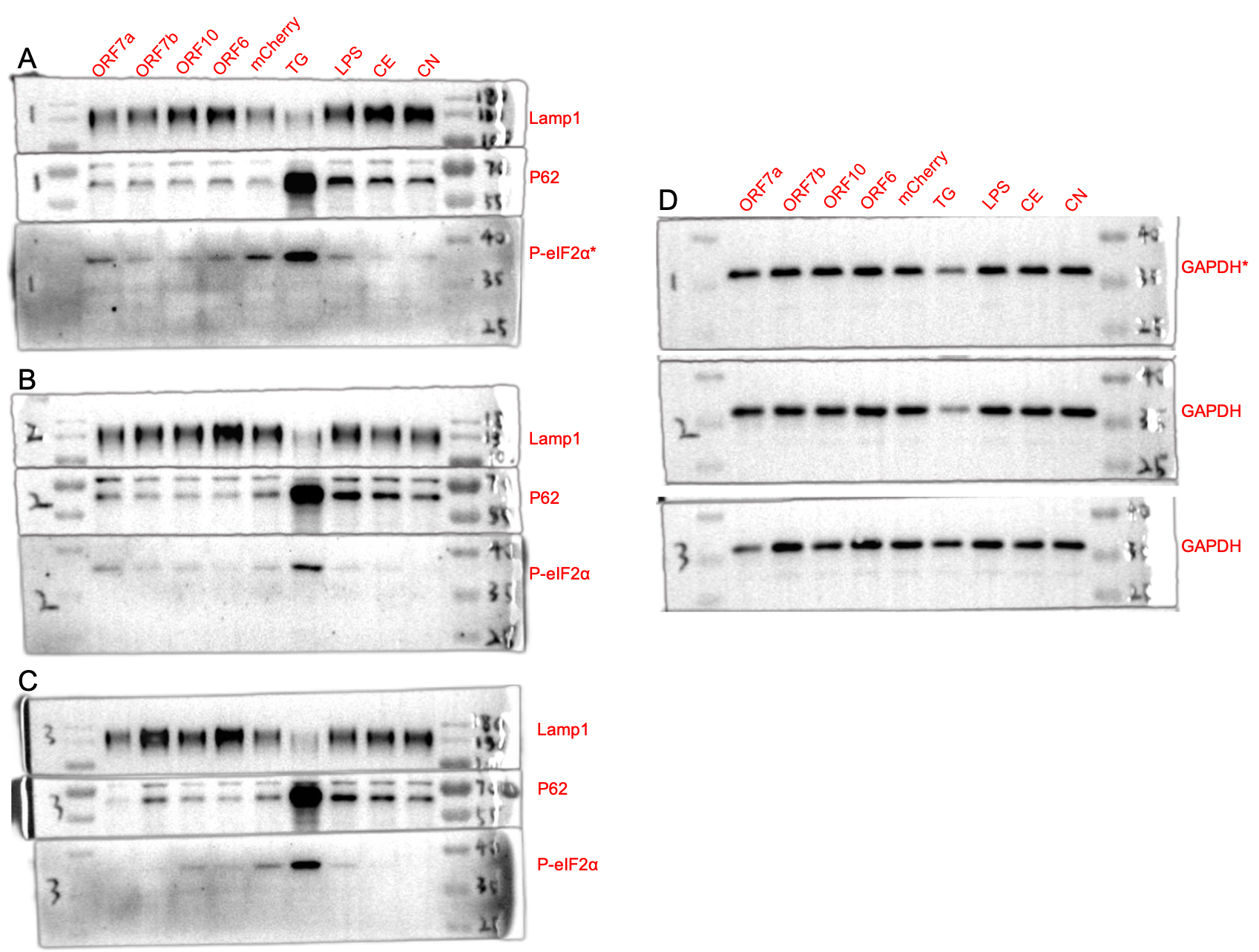
**Blot 5:** the western blot was repeated three times (A-C) for quantification. The bands of P-eIF2α and GAPDH in figure 6 are indicated in A and D. (**_*_**) indicates representative bands shown in figure 6. Due to the similar size of GAPDH and P-eIF2α, the membrane was stripped and re-incubate for P-eIF2α after GAPDH blotting.

**Blot 6:** the western blot was repeated four times (A-D) for quantification. The bands of P65, ATF4 and GAPDH in figure 6 are indicated in B. (**_*_**) indicates representative bands shown in figure 6.


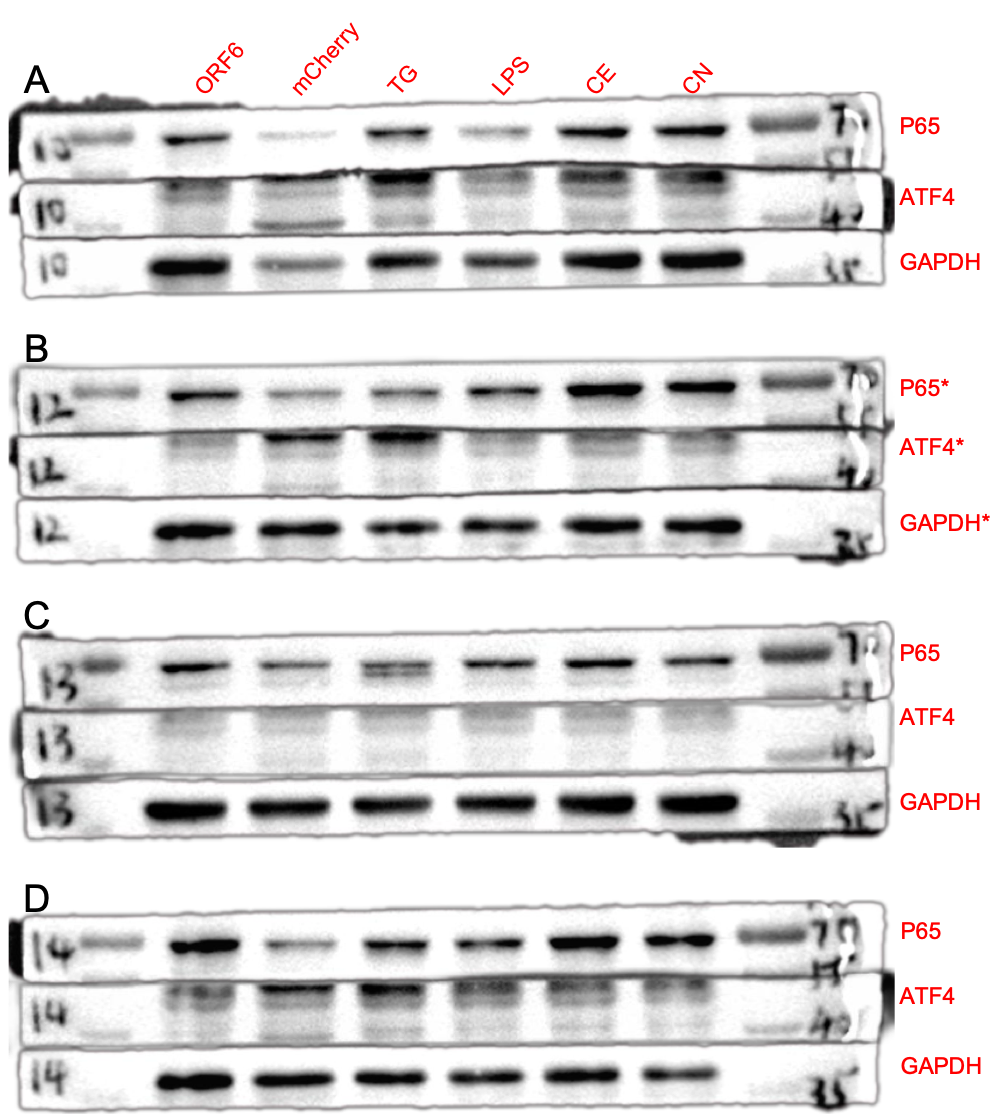


**Blot 7:** the western blot was repeated four times (A-D) for quantification. The bands of P-P65 and GAPDH in figure 6 are indicated in C. (**_*_**) indicates representative bands shown in figure 6.


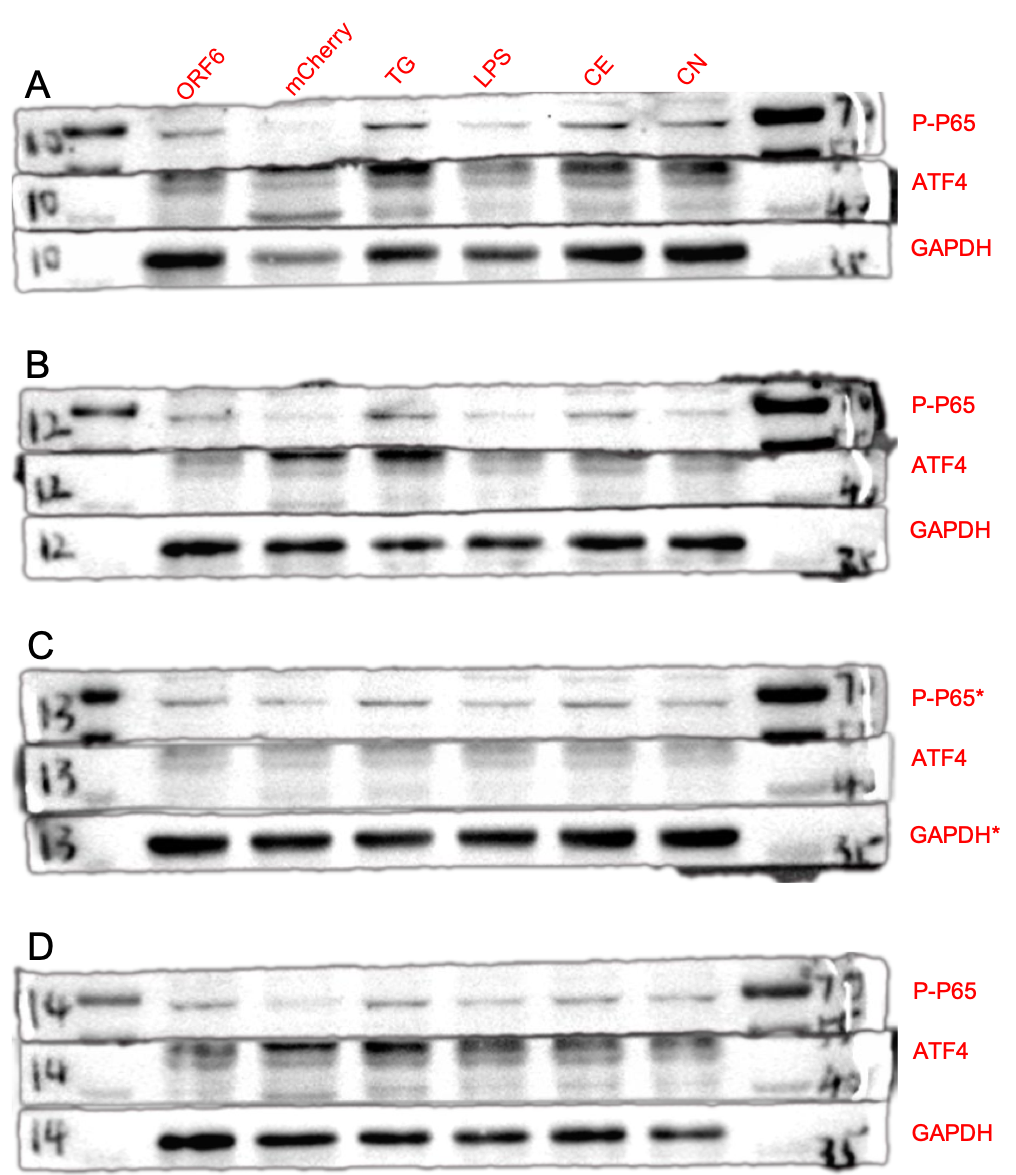

Supplement: Supplementary file 2 — Supplementary Information 2. [file 41598_2025_10013_MOESM2_ESM.docx]
